# A global annual fractional tree cover dataset during 2000–2021 generated from realigned MODIS seasonal data

**DOI:** 10.1038/s41597-024-03671-9

**Published:** 2024-08-01

**Authors:** Yang Liu, Ronggao Liu, Jilong Chen, Xuexin Wei, Lin Qi, Lei Zhao

**Affiliations:** 1 State Key Laboratory of Resources and Environmental Information System, Institute of Geographic Sciences and Natural Resources Research, Chinese Academy of Sciences, Beijing, 100101 China; 2 University of Chinese Academy of Sciences, Beijing, 100049 China

**Keywords:** Forestry, Forest ecology

## Abstract

Fractional tree cover facilitates the depiction of forest density and its changes. However, it remains challenging to estimate tree cover from satellite data, leading to substantial uncertainties in forest cover changes analysis. This paper generated a global annual fractional tree cover dataset from 2000 to 2021 with 250 m resolution (GLOBMAP FTC). MODIS annual observations were realigned at the pixel level to a common phenology and used to extract twelve features that can differentiate between trees and herbaceous vegetation, which greatly reduced feature dimensionality. A massive training data, consisting of 465.88 million sample points from four high-resolution global forest cover products, was collected to train a feedforward neural network model to predict tree cover. Compared with the validation datasets derived from the USGS circa 2010 global land cover reference dataset, the R^2^ value, MAE, and RMSE were 0.73, 10.55%, and 17.98%, respectively. This dataset can be applied for assessment of forest cover changes, including both abrupt forest loss and gradual forest gain.

## Background & Summary

Forests play an indispensable role in terrestrial ecosystems, providing essential resources and habitats for humans and wildlife. They serve a series of ecosystem services such as biodiversity conservation, climate regulation, and soil and water preservation. As the most substantial terrestrial carbon sink, forests are widely recognized as a key option for nature-based solutions for climate change mitigation^1,2^. In recent decades, forests have experiencing substantial changes with intensified human activities and global environmental changes. Considerable forest losses have been incurred by various factors, such as deforestation, wildfires, shifting agriculture, and urbanization^3^. Conversely, gains in forest cover have also been observed due to afforestation initiatives, forest conservation efforts, and natural growth of trees^4,5^. Additionally, the density of forests generally varies across different forest types, geographical regions, and succession stages. These variations in forest cover can have profound impacts on the structure and function of forest ecosystems^6^. The monitoring of forest cover state and its temporal dynamics is of great significance for forest management, carbon and water cycle modelling, and the assessment of environmental initiatives implementation.

Forest cover mapping has been one of the main applications of satellite remote sensing since the 1970s^7^. Many datasets depicting forest cover have been generated from optical satellites data such as AVHRR, MODIS, Landsat, and Sentinel-2, as well as from Synthetic Aperture Rader (SAR) data from instruments including PALSAR and Sentinel-1. These datasets typically fall into one of two categories, namely categorical forest or land cover and fractional tree cover. Categorical datasets classify areas as forests where are dominated by trees with the coverage exceeding a certain threshold. Examples of categorical forest cover datasets include the PALSAR Forest/Non-Forest Map (FNF)^8^ and conventional land cover maps such as GlobeLand30^9^ and ESA WorldCover^10^. However, this approach does not fully capture the variations of forest density^11^. For example, two regions with disparate tree covers, such as 30% and 90%, could be indiscriminately classified as the same forest class in such categorical maps. This limitation would hamper its ability to accurately characterize the heterogeneity of forest landscapes and potentially lead to an underestimation of forest cover dynamics that do not involve alterations in land cover types^12^.

Fractional tree cover enables a finer depiction of forest density by quantifying the percentage of a pixel covered by trees. Several global tree cover percentage products have been developed using satellite observations from MODIS, AVHRR, Landsat, and PROBA-V, with spatial resolutions ranging from 30 m to 5 km and temporal resolutions from annual to decadal updates. These products include the MODIS Vegetation Continuous Fields (VCF)^13^, AVHRR VCF^14^, Global Forest Change (GFC) tree cover^15^, Copernicus Global Land Service Land Cover Map products (CGLS-LC100)^16^, Global Forest Cover Change Tree Cover (GFCC30TC) dataset^17^, and the global tree cover percentage map by Kobayashi *et al*.^18^. These products are mostly generated based on machine learning algorithms, such as supervised regression tree and random forest regression models. Phenological metrics extracted from multi-temporal satellite observations are utilized as input features of these models for predicting tree cover percentage. To enhance the differentiation of trees, tens or even hundreds of metrics are often employed in global tree cover mapping. For example, 68, 735, 113, 183, and over 270 metrics were used for MODIS VCF, AVHRR VCF, GFC, CGLS-LC100, and Kobayashi maps, respectively. In terms of training data, training samples for these models are typically generated by aggregating high-resolution land cover maps from specific regions or through visual interpretation of high-resolution satellite imagery at selected sites. For instance, MODIS VCF’s training data were derived by aggregating the land cover classification results across more than 250 Landsat scenes. Kobayashi and colleagues created their training dataset through visual interpretation of Google images at 3086 sites, supplemented by simulation. While the CGLS-LC100 training data were collected by visually interpreting land cover types from Google images at 10 m resolution subpixels, and then aggregated to tree cover fraction estimates in 100 m PROBA-V grid. However, the use of an excessive number of input features can lead to the so-called “curse of dimensionality”, potentially resulting in model overfitting and increasing the possibilities of prediction error^19^. Moreover, the limited geographical coverage of training data would restrict its representativeness, thereby amplifying the uncertainty of tree cover estimates^20^. It has been reported that some existing tree cover percentage products suffer from inter-annual instability and discontinuity in their estimations^21,22^, which makes it hard to determine reliable trends in forest cover change analyses^23^.

This paper presents the GLOBMAP Fractional Tree Cover dataset (GLOBMAP FTC), which contains global annual fractional tree cover data at a spatial resolution of 250 m for the period from 2000 to 2021. We tried to improve tree cover estimation from two key steps of the machine learning model employed, including input features extraction and training data collection. For input feature, we extracted twelve discriminative features from realigned annual MODIS observations based on the different shape of the spectral seasonal curves between trees and herbaceous vegetation. For the training dataset, we generated a training data comprising 465.88 million sample points from four existing high-resolution forest cover products across the globe. A neural network model was calibrated and applied to predict tree cover estimates from MODIS data. This new dataset can support for monitoring of forest cover change and facilitate forest management practices.

## Methods

### Algorithm general framework

**Fig. 1:**
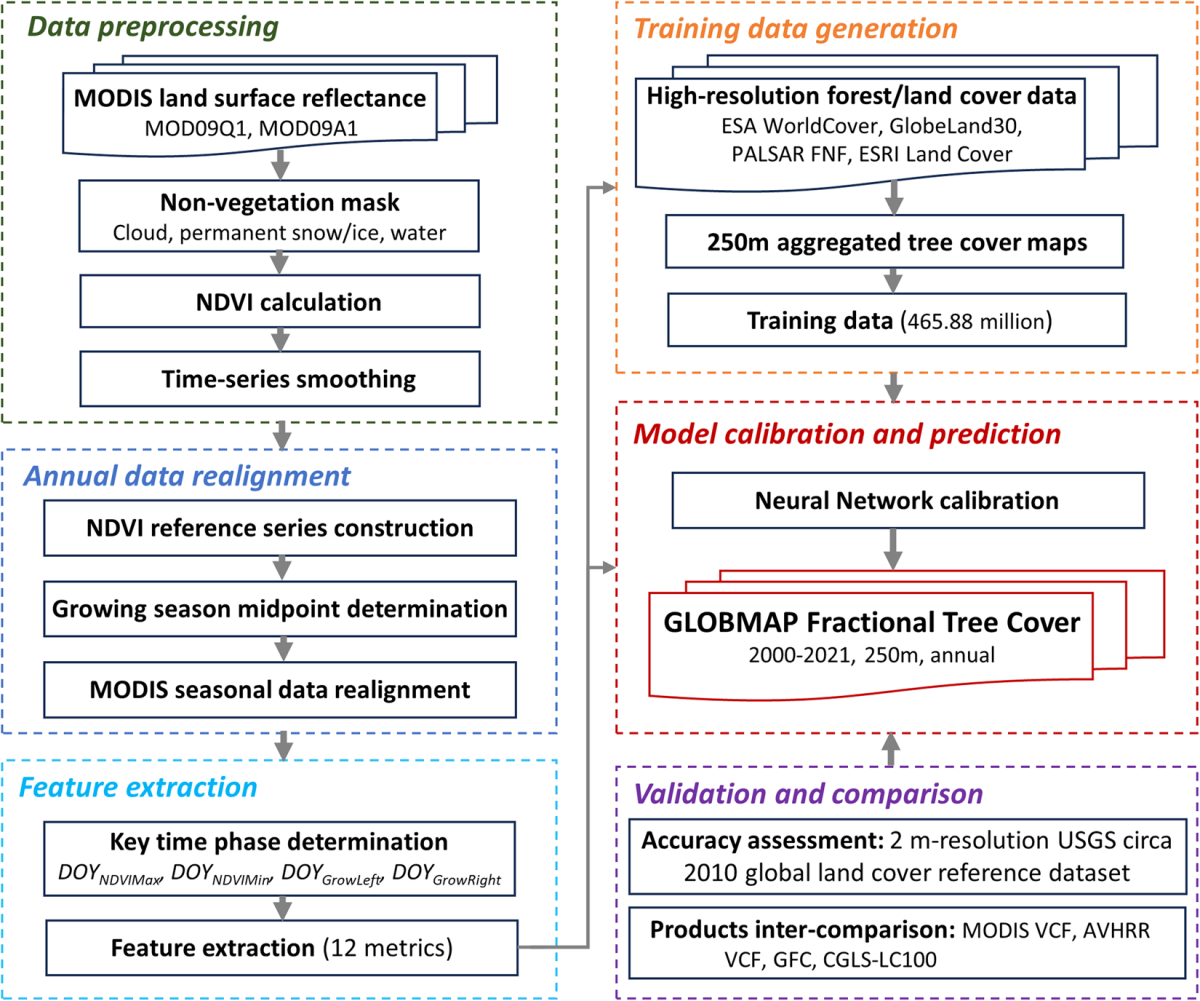
The workflow used to generate GLOBMAP Fractional Tree Cover dataset.

This study extracts fractional tree cover based on the distinctive spectral seasonal profiles of trees and herbaceous vegetation. We find that the satellite greenness of trees typically exhibits a sustained plateau during the middle period of the growing season, while that of herbaceous vegetation presents a downward-facing parabolic curve^24^. This contrast is related to the growth dynamics of these vegetation types. The foliage of trees usually experiences rapid growth and senescence within a brief period at the start and end of the growing season, whereas that of herbaceous vegetation exhibits a more prolonged and gradual process of growth and senescence. By using the temporal phases when the difference between the two vegetation types is most pronounced, we can distinguish between them using a few number of features. In large-scale vegetation mapping, this becomes more complicated due to the considerable variability in the timing of the growing seasons across different geographical regions^25^. To eliminate the impact of these phenological discrepancies, we performed pixel-by-pixel realignment of the annual MODIS observations to make the midpoint of the growing seasons align with the center of the calendar year. Based on these realigned annual sequences, twelve features were extracted from observations of four key time phases for tree cover estimation. The training dataset was derived by aggregating and combining four publicly available forest cover products with spatial resolution ranging between 10 and 30 m. A feed-forward neural network was trained using the training dataset to predict tree cover from the MODIS observations. The workflow of the proposed tree cover extraction algorithm was illustrated in Fig. 1.

### Data preprocessing

MODIS land surface reflectance products (MOD09Q1^26^ and MOD09A1^27^) were utilized to generate fractional tree cover maps. The MOD09Q1 product provides 8-day composite global land surface reflectance at spatial resolution of 250 m in the red and near-infrared (NIR) bands, which are critical for vegetation monitoring. While the MOD09A1 product contains similar 8-day composite data across a broader spectral range, including MODIS bands 1–7 at 500 m resolution. The Normalized Difference Vegetation Index (NDVI) was calculated with 250 m resolution every 8 days from the land surface reflectance in red and NIR bands from the MOD09Q1 product. The short-wave infrared band at 2.1 μm (SWIR2.1, corresponding to MODIS band 7) from the MOD09A1 product was also used in the proposed algorithm due to its high sensitivity to variations in forest density^28^, making it valuable for tree cover estimation. The SWIR2.1 band reflectance was resampled to 250 m resolution to match the NDVI data and reflectance in red and NIR bands. Water bodies and permanent snow and ice were masked using the state flag in MODIS products, and clouds were identified and masked using an enhanced cloud detection method^29^. The gaps in NDVI time series were filled and smoothed using the locally adjusted cubic spline capping (LACC) approach^30^.

### Realignment of annual MODIS observations

**Fig. 2:**
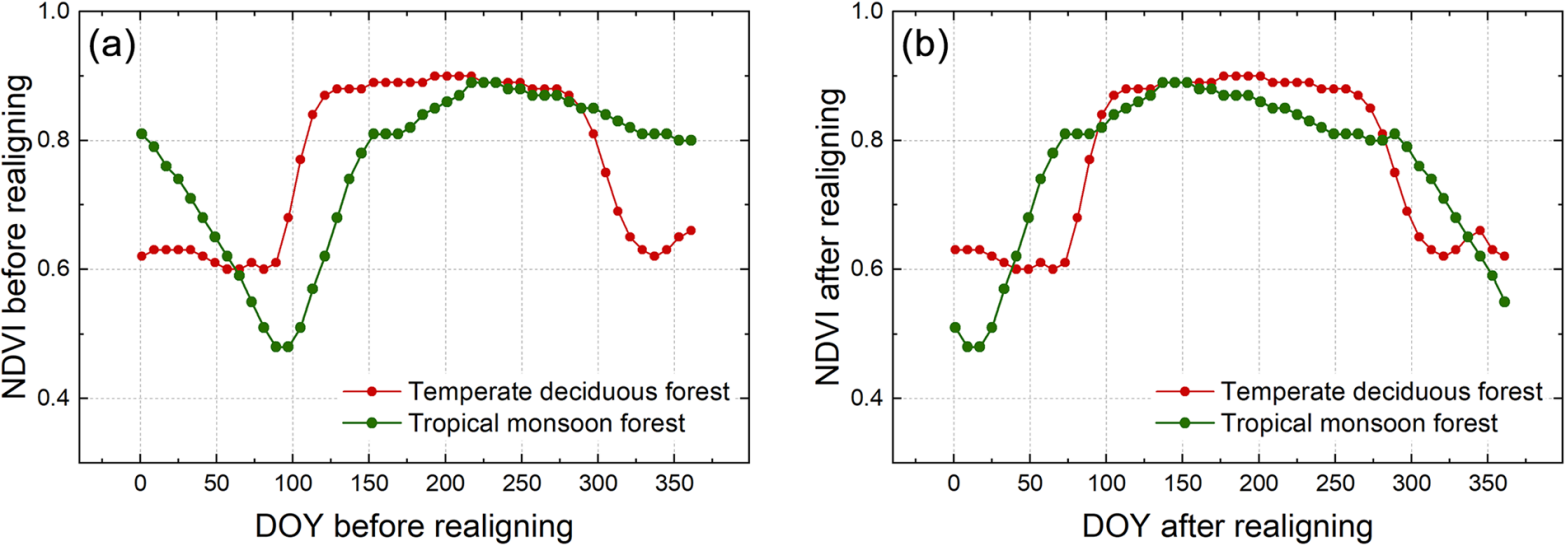
Seasonal profiles of NDVI series before and after realigning. NDVI series (**a**) before and (**b**) after realigning for the temperate deciduous forest in the USA (78.76°W, 35.85°N) and tropical monsoon forest in India (78.81°E, 15.62°N) in 2021.

Annual MODIS observations were realigned to a common phenology using reconstructed NDVI data. For each pixel, the NDVI reference seasonal series were generated by averaging NDVI values over a 22-year period (2000–2021) at 8-day intervals. We determined the midpoint date of the growing season from this NDVI reference seasonal series and moved to the center of the calendar year, specifically day of year (DOY) 185–192. Other dates were then shifted accordingly. Those exceeding beyond the temporal boundary of the current year after data shifting were moved to the vacant period within this year to construct a complete annual spectral curve. The growing season was determined by selecting a sequence of consecutive NDVI values exceeding half of the pixel’s annual NDVI amplitude, and the central date of this sequence was identified to represent the midpoint of the growing season. In areas where multiple NDVI peaks exceeded half of the annual NDVI amplitude within a year, the mean daytime land surface temperature was calculated for each identified growing season period using the MODIS 8-day land surface temperature/emissivity products (MOD11A2^31^), and the peak exhibiting the highest mean temperature was chosen as the primary growing season for sequence realignment purpose. For evergreen vegetation, which was determined by the difference between maximum NDVI and minimum NDVIs of less than 0.2, the eighteen highest NDVI values within the year were utilized to identify the midpoint of the growing season. Figure 2 shows an example of annual sequence realignment for two forest sites, including tropical monsoon rainforest and temperate deciduous forest. Before realignment, the NDVI seasonal curves of the two sites all presented NDVI plateau, but these curves showed different shapes due to the disparate timings of the growing season across these sites. After realignment, the NDVI curves demonstrated similar shapes, with each site’s growing season centered within the year. This realignment process harmonizes the spectral consistency for areas with similar tree cover across various forest types and geographic regions, which helps to extract highly distinguishable features for tree cover estimation.

### Feature extraction

**Fig. 3:**
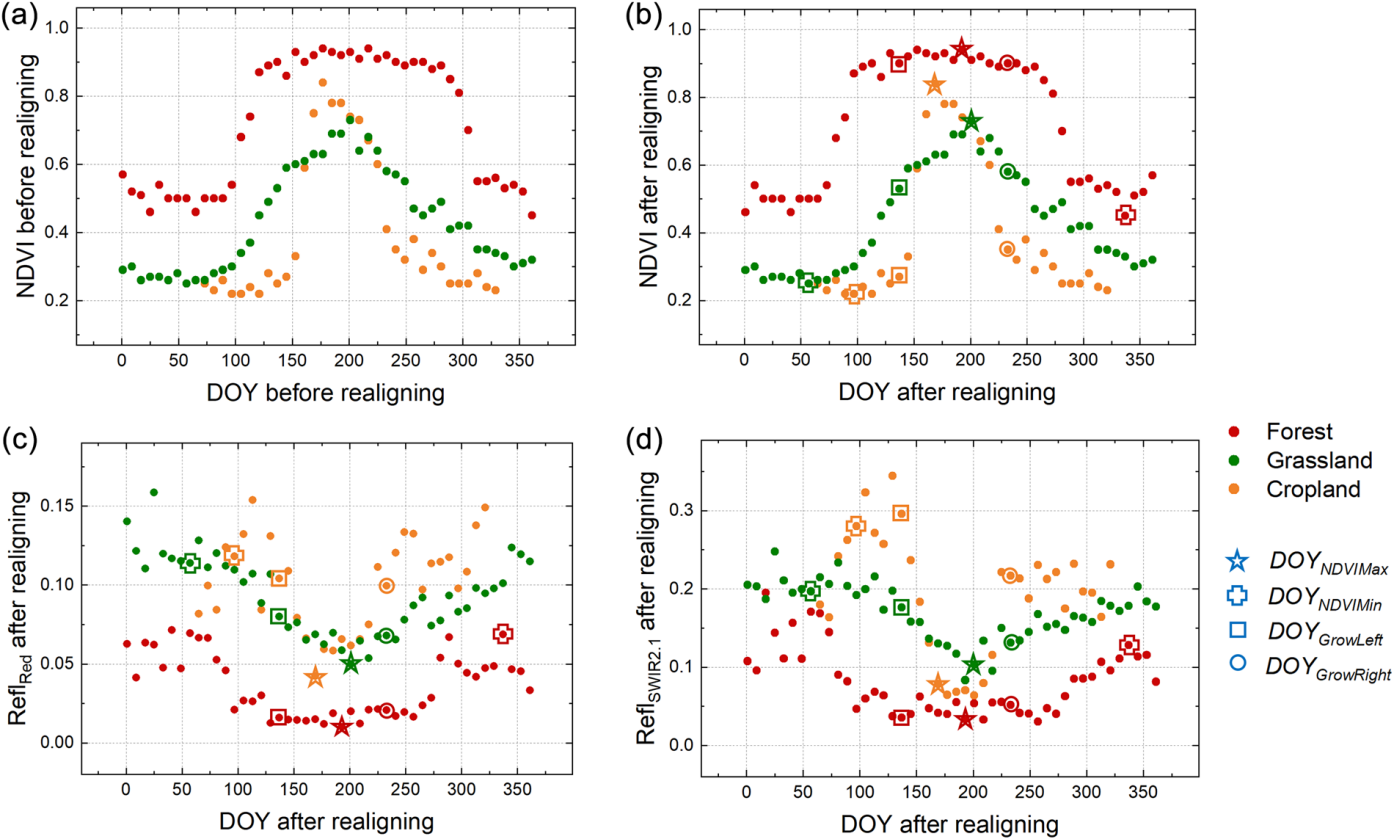
Seasonal profiles of NDVI and spectral reflectance in the red and SWIR2.1 bands and the extracted features at BELMANIP sites covered with different vegetation types. These include Fairystone in the USA (80.09°W, 36.77°N) covered with deciduous broadleaf forests, Alberta in the Canada (113.90°W, 51.70°N) covered with croplands, and ARM/CARTShilder in the USA (96.86°E, 36.93°N) covered with grasslands. Seasonal series of (**a**) NDVI before realigning; (**b**) NDVI after realigning; (**c**) Red band reflectance after realigning; (**d**) SWIR2.1 band reflectance after realigning.

Twelve metrics were extracted from the realigned annual MODIS observations to serve as input features for the generation of fractional tree cover dataset. These features were derived from observations spanning four key temporal phases that exhibited substantial discriminatory between trees and herbaceous vegetation. These phases included the DOY corresponding to the annual maximum NDVI (*DOY*_*NDVIMax*_), the annual minimum NDVI (*DOY*_*NDVIMin*_), and two phases occurring approximately one and a half months before (*DOY*_*GrowLeft*_) and after (*DOY*_*GrowRight*_) the identified midpoint date of the growing season. The *DOY*_*NDVIMax*_ was determined by directly finding the highest NDVI value within the year, while the *DOY*_*NDVIMin*_ was identified by finding the lowest NDVI value of the year using the brown vegetation index^32^. Given that the midpoints of the growing season in the realigned sequences were all centered to DOY185–192, the *DOY*_*GrowLeft*_ and *DOY*_*GrowRight*_ corresponded to DOY137–144 and DOY233–240, respectively. At these four time phases, the corresponding NDVI value, red band reflectance, and SWIR2.1 band reflectance were extracted from the realigned annual MODIS observations, yielding twelve features in total. Figure 3 illustrated the realigned spectral seasonal series and the extracted features for three Benchmark Land Multisite Analysis and Intercomparison of Products (BELMANIP) sites, including forests, grasslands, and croplands. These seasonal series were constructed by calculating the mean of valid observations that were free from cloud and snow from 2019 to 2021. The extracted features from forests differ notably from those of croplands and grasslands, with forests typically exhibiting higher NDVI values and lower reflectance in both the red and SWIR2.1 bands during the specified temporal phases in contrast to grasslands and croplands. Notably, the spectrum of dense croplands may be close to that of forests at *DOY*_*NDVIMax*_, but the features from the other three phases provide a means for their differentiation.

### Training data collection

The training data were generated from four global high-resolution forest cover products for the year 2020, namely ESA WorldCover, GlobeLand30, PALSAR FNF, and ESRI Land Cover. ESA WorldCover contains global land cover maps with 10 m resolution for the years 2020 and 2021 derived from Sentinel-1 and Sentinel-2 data, with the user’s and producer’s accuracies reported of 80.8% and 89.9% for the tree cover class for the 2020 maps^10^. GlobeLand30 provides global 30 m resolution land cover maps for the years 2000, 2010, and 2020 created from Landsat-like images, with the user’s accuracy reported exceeding 83% for forest class^9^. The PALSAR FNF product includes global annual forest/non-forest maps at 25 m resolution since 2007 based on L-band PALSAR/PALSAR-2 SAR data, with an overall accuracy reported to be over 85%^8^. Lastly, ESRI Land Cover provides global annual land cover maps with 10 m resolution for the period from 2017 to 2022 based on Sentinel-2 data, with the trees class reporting user’s and producer’s accuracies of 90.35% and 91.07%, respectively^33^. For the ESA WorldCover, GlobeLand30, and PALSAR FNF products, the forest or tree cover classes are defined as areas where trees are dominant with a coverage exceeds 10%. Moreover, the PALSAR FNF product requires a minimum contiguous area of tree cover greater than 0.5 ha. In the case of the ESRI Land Cover product, the trees class is delineated based on the presence of significant clusters of tall dense vegetation, with tree heights of 15 m or taller.

**Fig. 4:**
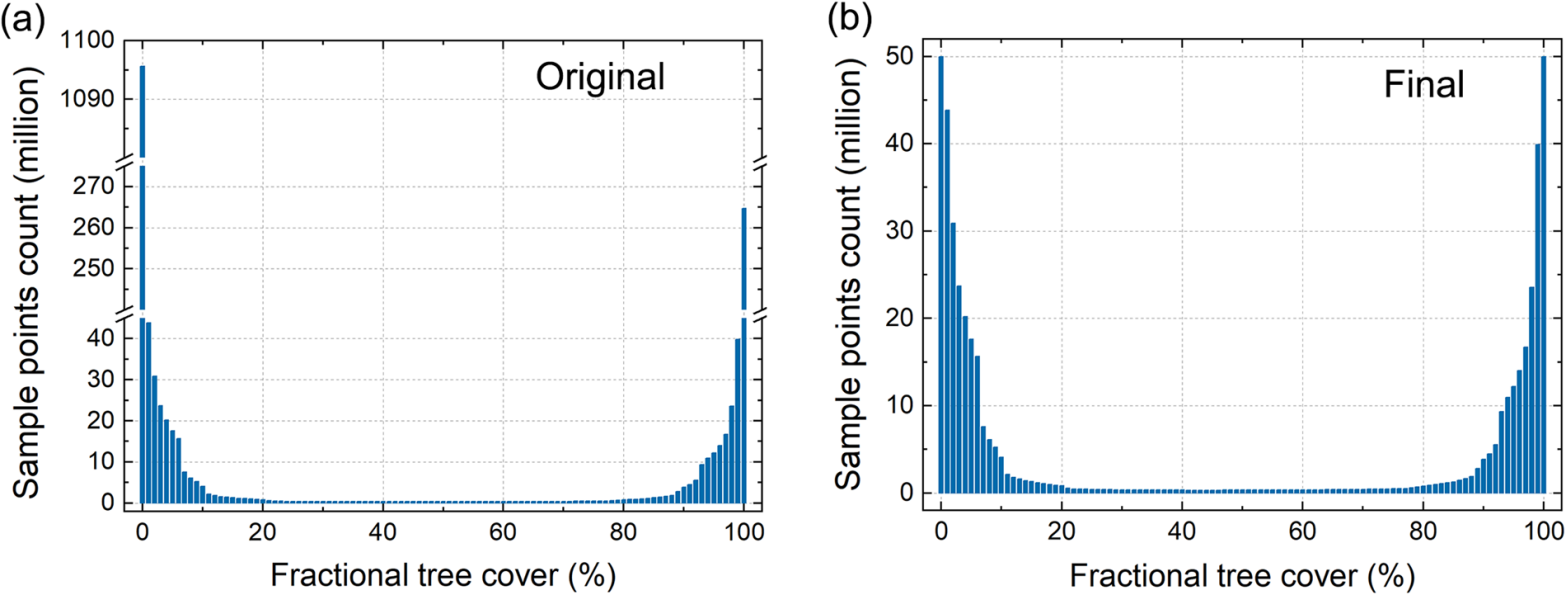
Histograms of reference tree cover samples for training data collection. (**a**) Histogram showing the distribution of original sample points; (**b**) histogram of final sample points after implementing sample balancing.

The fractional tree cover reference maps were generated by aggregating and combining the 2020 maps from the above four products. The four categorical forest maps were aggregated to the fractional tree cover maps by calculating the proportion of pixels classified as forest/tree class within each 250 m grid. These maps were converted to a Sinusoidal projection to match with MODIS data. Then, the reference map (*TC*_*RefMean*_) was then produced by averaging the four aggregated tree cover maps, and the mean absolute error (*MAE*_*Ref*_) of each individual aggregated tree cover map relative to the *TC*_*RefMean*_ was also computed to assess its deviation. The training data were then collected from the *TC*_*RefMean*_ map across the globe. Areas where the *MAE*_*Ref*_ exceeded 10% were excluded to eliminate the impacts of possible uncertainties in these categorical forest/land cover maps. These areas were predominantly characterized by sparse forests and complex landscapes, such as low tree-covered savannas and northern boreal forests. Water bodies, permanent snow and ice, and barren lands were also excluded using the MODIS land cover type products (MCD12Q1). Areas with fewer than 25 clear-sky MODIS observations in a year or with a growing season of less than three months were also excluded from the training dataset. More than 1.72 billion sampling points were collected from the *TC*_*RefMean*_ map. Up to 63.44% (approximately 1095.60 million sample points) were found in the 0% tree cover bin, and 15.33% (264.72 million sample points) were concentrated in the 100% tree cover bin. While the counts for other tree cover bins (1% to 99% tree cover) varied from 0.34 million to 43.85 million (Fig. 4a). Such great disparities in sample distribution across different tree cover densities may lead to sample imbalance during the training of machine learning models. This may result in the model exhibiting superior performance for tree cover categories with a large number of training samples, while demonstrating inferior performance for categories with a small number of samples. To balance the samples across different tree cover densities, 50 million sample points were randomly selected from both the 100% and 0% tree cover categories, respectively. All sample points from other density categories were retained. These sample points were then paired with corresponding input features extracted from the MODIS observations in 2020 to form the training dataset. The resulted final training dataset comprised 465.88 million sample points, with the minimum count of sample points for each 1% tree cover bin alone no fewer than 330,000 (Fig. 4b).

### Neural network training and fractional tree cover maps generation

A feedforward neural network model was adopted to generate fractional tree cover maps. The neural network model consisted of twelve neurons for the input layer, corresponding to the twelve input features. The hidden layer of the model contained five layers, comprising 400, 200, 100, 50, and 25 neurons in each layer, respectively. The Rectified Linear Unit (ReLU) algorithm was utilized as the activation function^34^. The output layer comprised a single neuron, which was dedicated to the target variable of the fractional tree cover. The neural network model was trained using the aforementioned training dataset derived from four existing global forest cover datasets for the year 2020. Then, the calibrated model was applied to produce global fractional tree cover maps for the period from 2000 to 2021 from the realigned MODIS observations. These maps could represent an optimized average of the four global forest cover maps. Areas identified as water bodies and permanent snow/ice were masked using the MODIS land cover type products (MCD12Q1). Additionally, regions exhibiting an annual maximum NDVI value below 0.35 were marked as non-tree covered areas, and their tree cover estimates were assigned a value of 0%.

## Data Records

The GLOBMAP Fractional Tree Cover dataset is publicly accessible through the Zenodo repository at 10.5281/zenodo.10589730^35^. This dataset contains global tree cover data spanning a 22-year period from 2000 to 2021, with the temporal and spatial resolutions of yearly and 250 m respectively. It is provided by 296 to 323 tiles for each year, aligning with the MODIS standard tiling system. Each tile covers an area of 1200 km × 1200 km, comprising of a matrix of 4800 rows by 4800 columns. The data are stored in the Sinusoidal projection, adhering to the Geotiff format for ease of use and compatibility. The files are designated with the naming structure “GLOBMAPFTC.AYYYY001.hHHvVV.V01.tif”, where “YYYY” represents the year of the data, while “HH” and “VV” indicate the horizontal and vertical tile indices respectively, aligning with the MODIS standard tiling nomenclature. The data layer within the file, named “TreeCover”, presents the fractional tree cover values. These values range from 0 to 100, with a scale factor of 1.0 and the unit specified as percentage (%). The dataset can be visualized and analysed with GIS remote sensing software, such as ArcGIS and ENVI.

## Technical Validation

The evaluation of the proposed method and the resulting fractional tree cover maps included three aspects: (1) accuracy assessment of the estimation results using 2 m resolution United States Geological Survey (USGS) circa 2010 global land cover reference dataset; (2) comparative analysis with other tree cover percentage products; (3) application of the dataset in monitoring forest cover changes at global and site scales.

### Accuracy assessment using USGS circa 2010 global land cover reference dataset

**Fig. 5:**
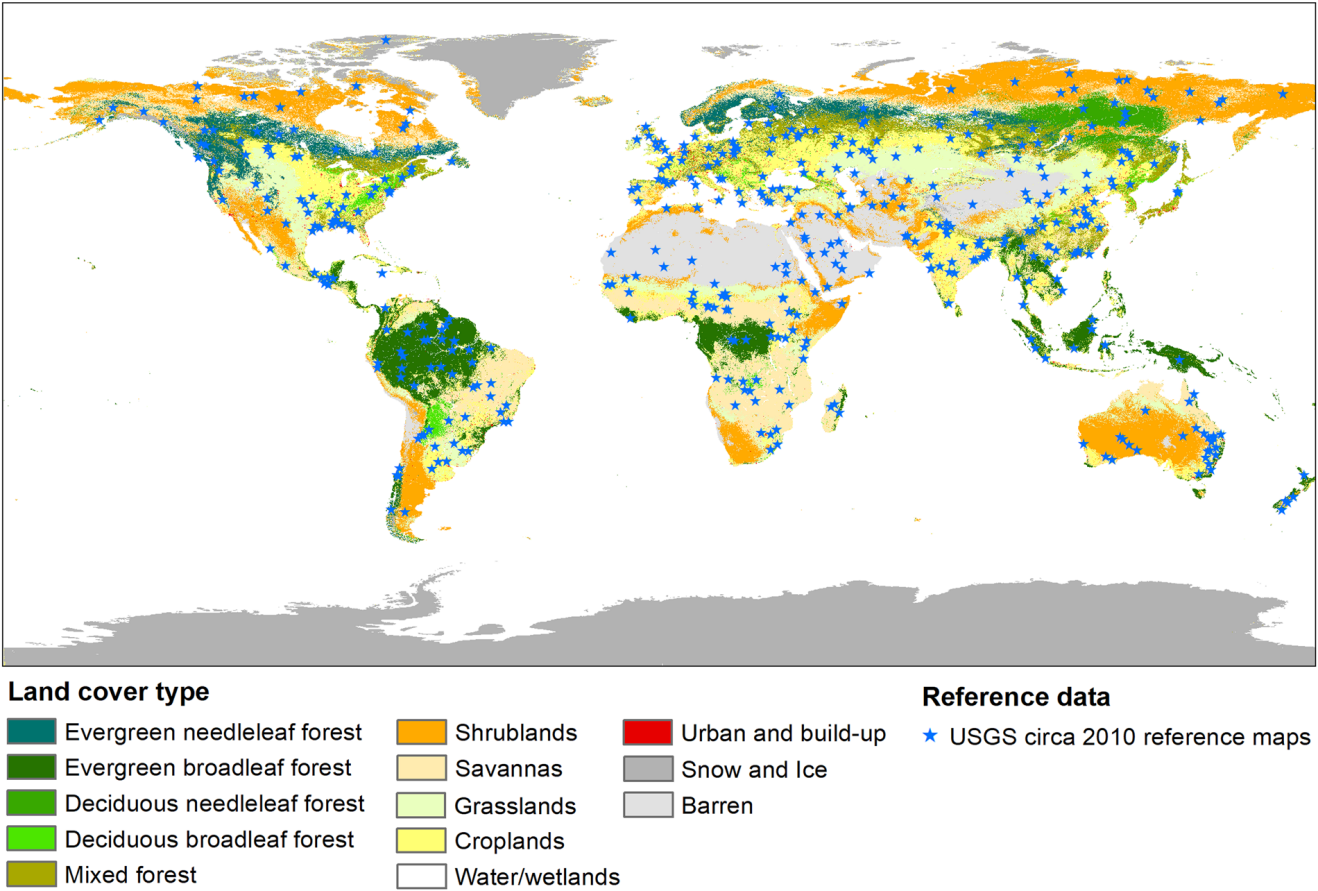
Distribution of fractional tree cover reference maps for dataset validation. The reference maps were generated from USGS circa 2010 global land cover reference dataset (*N* = 475). The land cover maps used MODIS land cover type products MCD12Q1.

The USGS provided a global land cover reference dataset circa 2010 at 475 sample blocks at 2 m resolution^36^ (Fig. 5). These sample blocks were selected globally through stratified random sampling within a sample frame that was stratified by a modified Köppen Climate/Vegetation classification coupled with population density^37^. Each sample block was 5 km × 5 km in size. Categorical land cover maps were generated by classifying sub-meter-resolution imageries from commercial satellites, such as QuickBird, IKONOS, and WorldView. The maps contained five land cover types, including tree, other vegetation, water, barren, and ice & snow. These thematic land cover maps were aggregated to the tree cover percentage maps at 250 m resolution by calculating the proportion of pixels classified as tree class within the 250 m grid. Pixels labelled as no data, cloud, and shadow were excluded in reference data generation. These reference maps were converted from their original Universal Transverse Mercator (UTM) projection to Sinusoidal projection, aligning with the GLOBMAP FTC dataset. We extracted the estimation results that matched the reference data in both spatial and temporal terms from the GLOBMAP dataset. The paired estimated and reference fractional tree cover values were used to calculate accuracy metrics, including the Mean Absolute Error (MAE), Root Mean Squared Error (RMSE), Mean Error (ME), and the coefficient of determination (R^2^). To mitigate the effects of positional misalignment between the two dataset that related to their different projections, both data were resampled to 500 m resolution, resulting in 62,022 reference sample points for the accuracy assessment. Considering that the sample blocks of the USGS circa 2010 global land cover reference dataset were not allocated proportionally to the strata areas, the MAE, RMSE, and ME were calculated using following equations to account for the unequal inclusion probabilities between different strata in the validation process against the USGS reference data^37,38^.

1 $${MAE}=\frac{{\sum }_{i=1}^{n}{\omega }_{i}|{{pre}}_{i}-{{ref}}_{i}|}{{\sum }_{i=1}^{n}{\omega }_{i}}$$

2 $${RMSE}=\sqrt{\frac{{\sum }_{i=1}^{n}{{\omega }_{i}({{pre}}_{i}-{{ref}}_{i})}^{2}}{{\sum }_{i=1}^{n}{\omega }_{i}}}$$

3 $${ME}=\frac{{\sum }_{i=1}^{n}{\omega }_{i}({{pre}}_{i}-{{ref}}_{i})}{{\sum }_{i=1}^{n}{\omega }_{i}}$$

where ${{pre}}_{i}$ is the estimated fractional tree cover, and ${{ref}}_{i}$ is the reference tree cover derived from USGS circa 2010 global land cover reference dataset at a pixel *i* in a sample of size *n*. ${\omega }_{i}$ is the estimation weight, the inverse of inclusion probability, for sample pixel *i*.

4 $${{\rm{\omega }}}_{i}=\frac{{K}_{h}}{{k}_{h}}$$

Where ${K}_{h}$ and ${k}_{h}$ are the population size and sample size for stratum *h*, respectively (see Olofsson *et al*.^37^ for the values of ${K}_{h}$ and ${k}_{h}$).

**Fig. 6:**
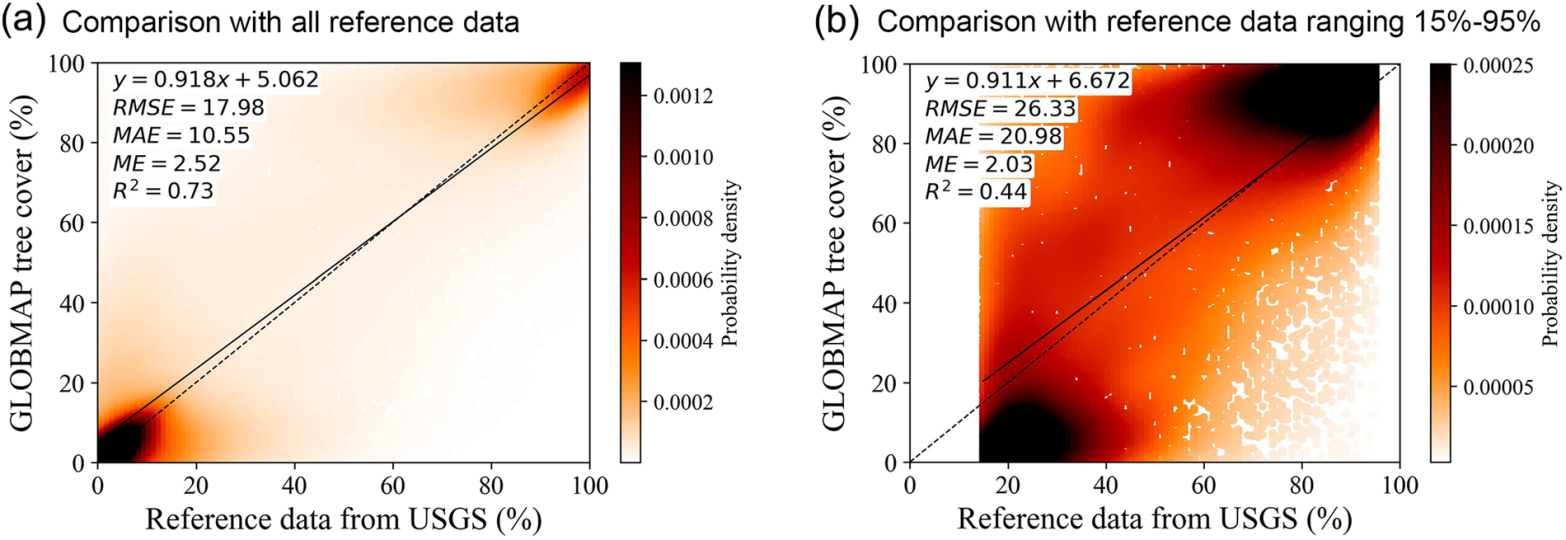
Accuracy assessment of GLOBMAP tree cover estimates against reference data derived from USGS circa 2010 global land cover reference dataset. Scatter plot for comparison with (**a**) all reference data (*N* = 62,022); and (**b**) reference data with tree cover percentages ranging from 15% to 95% (*N* = 24,146).

Figure 6a presented the scatter plot of the validation against all reference sample points derived from the USGS circa 2010 global land cover reference data (*N*=62,022). A positive correlation was presented between the estimated tree cover and reference data, with an R^2^ value of 0.73. The linear regression line approximated to the ideal 1:1 line, with a slope of 0.918. The ME, MAE, and RMSE were 2.52%, 10.55%, and 17.98%, respectively. Notably, the majority of reference sample points were distributed below 15% and above 95% tree cover. To better assess the performance of the tree cover between 15% and 95%, we excluded sample points where tree cover percentages were either below 15% or above 95% from the reference dataset, as determined by the reference samples. The adjustment resulted in a modified reference dataset comprising 24,146 sample points, which was then used for further analysis (Fig. 6b). Compared with the USGS reference dataset with tree cover ranging from 15% to 95%, the metrics showed an R^2^ of 0.44, ME of 2.03%, MAE of 20.98%, and RMSE of 26.33%. The R^2^ value decreased and error metrics increased compared to the analysis utilizing all validation samples. This suggested that the GLOBMAP FTC dataset demonstrated better performance in areas with dense forests or non-forests, while uncertainties would increase in regions with sparse or medium-density forests. Our estimation results tended to underestimate tree cover in sparsely forested areas, particularly in those with tree cover between 15% and 30%. This underestimation was likely attributed to the limited ability of coarse MODIS observations to capture sparse and fragmented trees. Conversely, tree cover estimates for ranges between 60% and 70% might be overestimated, potentially related to the methodologies employed in generating the training dataset. The training data for this study were generated by aggregating and combining four high-resolution categorical forest/land cover products. Among them, the ESA WorldCover, GlobeLand30, and PALSAR FNF products defined the forest or tree classes as areas dominated by trees with tree cover exceeding 10%^8–10^. This could potentially lead to an overestimation of tree cover in the training data, and consequently in the model predictions. This overestimation was particularly likely to occur in sparsely forested areas. To alleviate the impact of this issue, we tried to eliminate sparse tree-covered areas from the training dataset by excluding areas where *MAE*_*Ref*_ exceeded 10%. Besides, the inclusion of the ESRI Land Cover product, which represented dense, tall vegetation, also contributed to this mitigation effort.

### Spatial distributions of derived global fractional tree cover

**Fig. 7:**
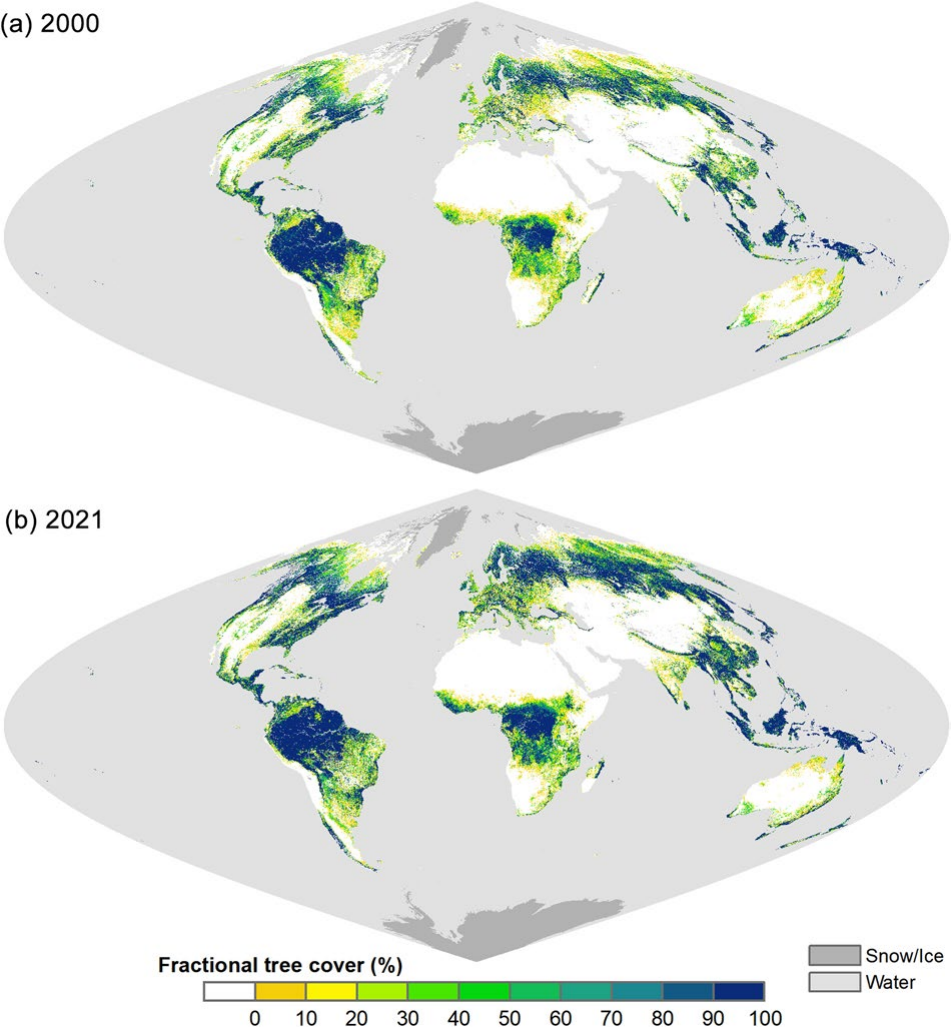
Global maps of GLOBMAP fractional tree cover. Tree cover map for (**a**) 2000 and (**b**) 2021.

Figure 7 illustrated the global distribution of fractional tree cover of the GLOBMAP dataset for the years 2000 and 2021. The results showed that the dataset could capture the distribution and density patterns of global forests. In densely forested areas, such as the tropical rainforests, dense temperate forests, and the southern extents of the boreal forests in the Northern Hemisphere, the estimated tree cover generally exceeded 90%. While the northern boreal forests, most temperate forests, and the forests in South America and Africa outside the tropical rainforests typically exhibited tree cover ranging from 40% to 90%. In savanna regions, the tree cover estimates were mostly between 10% and 70%, suggesting substantial heterogeneity within this biome. The tree cover was mostly below 10% or approximately 0% for croplands and grasslands, while it was 0% for barren lands. Compared with 2000, fractional tree cover increased across many regions globally in 2021. Notable tree cover gains were observed in southern China, the high latitudes of the Northern Hemisphere, and African savanna areas. A detailed analysis of the changes in global forest cover was presented in the subsequent sections of the paper.

### Comparative analysis with other tree cover percentage products

The GLOBMAP dataset was compared with four existing tree cover percentage products, including GFC percent tree cover^15^ (30 m resolution, 2000 and 2010), MODIS VCF^13^ (250 m resolution, 2000–2020), CGLS-LC100^16^ (100 m resolution, 2015–2019), and AVHRR VCF^14^ (0.05°resolution, 1982–2016 except 1994 and 2000). The GFC and CGLS-LC100 products were resampled to 250 m resolution by calculating the proportion of tree covered area within each 250 m grid to be consistent with the GLOBMAP and MODIS VCF datasets. For the comparison with AVHRR VCF, the GLOBMAP dataset was resampled to 0.05° resolution. The latter three products were converted to a Sinusoidal projection. To evaluate the consistency between the GLOBMAP dataset and the other tree cover products, the Mean difference (*MD*) was calculated using Eq. (5) with data from all available overlapping years. These periods included the years 2000 and 2010 for GFC, 2000–2020 for MODIS VCF, 2015–2019 for CGLS-LC100, and 2001–2016 for AVHRR VCF.

5 $${{MD}}_{i}=\frac{{\sum }_{y=1}^{Y}({{GLOBMAPFTC}}_{(i,y)}-{{CompareFTC}}_{(i,y)})}{Y}$$

Here ${{MD}}_{i}$ was the mean difference for each pixel *i*. ${{GLOBMAPFTC}}_{\left(i,y\right)}$ and ${{CompareFTC}}_{(i,y)}$ referred to the fractional tree cover values of GLOBMAP and datasets used for comparison for pixel *i* in year *y*, respectively. *Y* was the total number of years for comparative analysis.

**Fig. 8:**
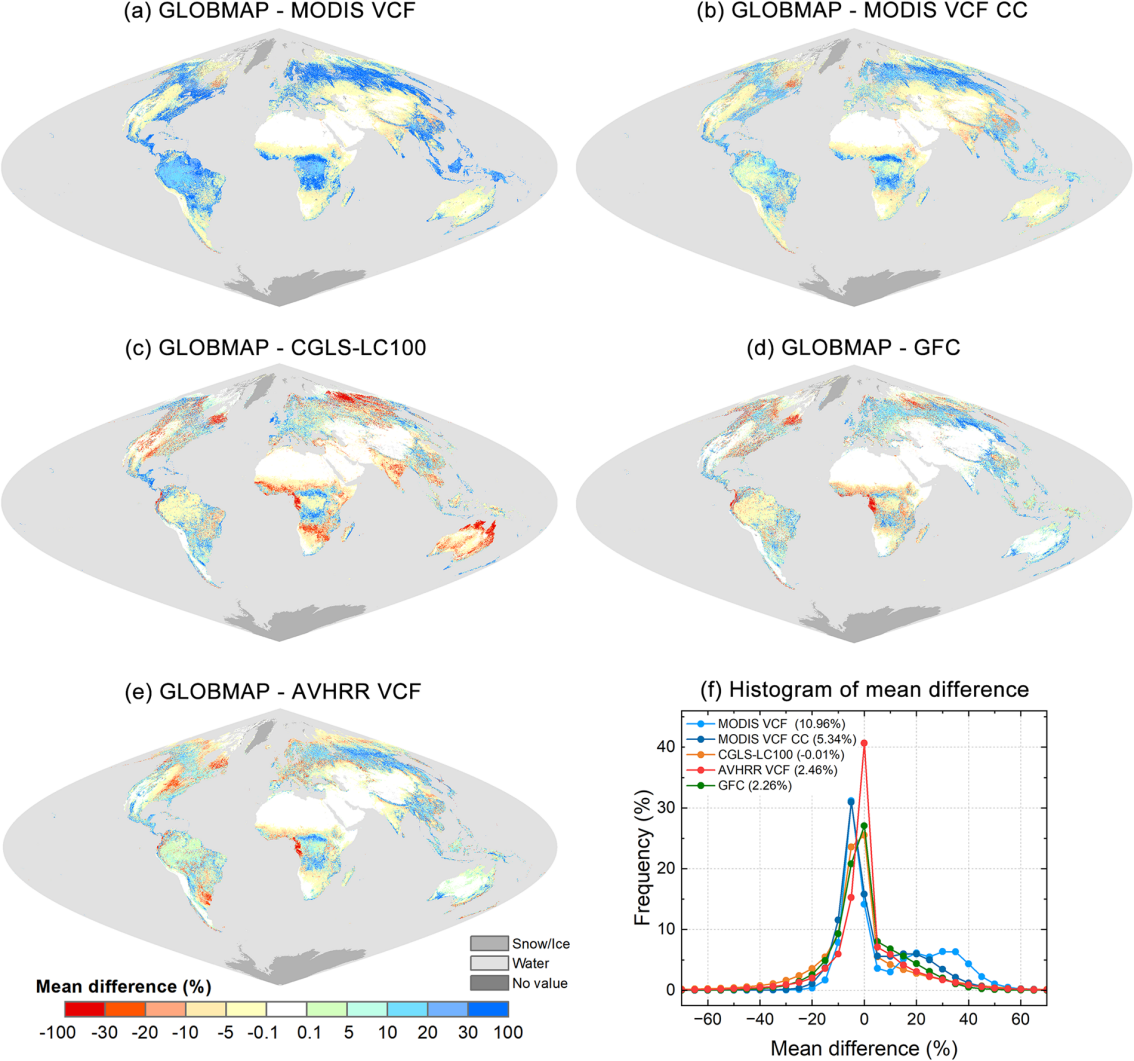
Comparison with other tree cover percentage products. Mean difference between GLOBMAP and (**a**) MODIS VCF during 2000–2020; (**b**) MODIS VCF tree crown cover during 2000–2020; (**c**) CGLS-LC100 during 2015–2019; (**d**) GFC for the years 2000 and 2010; (**e**) AVHRR VCF during 2001–2016; (**f**) histograms of mean difference over global vegetated areas for (**a**–**e**). The number in brackets in (**f**) was the global average value of mean difference over vegetated areas. The vegetated areas were determined using MODIS land cover type products (MCD12Q1).

Figure 8 displayed the global distribution of the *MD* values and their corresponding histograms over vegetated areas. The vegetated areas were determined using the MODIS land cover type products (MCD12Q1), with water bodies, permanent snow/ice, and barren lands excluded from the analysis. The results demonstrated that our estimates exhibited the smallest global-scale difference with the CGLS-LC100 product, with an average *MD* of −0.01% across global vegetated regions. The GLOBMAP FTC dataset presented relatively small discrepancies when compared with the widely used high-resolution GFC product, with a global average *MD* of 2.26% and most *MD* values within ± 5% in tropical rainforests. The difference with the coarse-resolution AVHRR VCF product was close to that of the GFC, with a global average *MD* of 2.46%. 74.40%, 82.62%, and 82.50% of vegetated pixels exhibited an *MD* of less than 15% for these three products. In contrast, a greater deviation was observed when comparing our estimates to MODIS VCF, with the global average *MD* reaching 10.96% over vegetated areas. This discrepancy was likely related to the definition employed by MODIS VCF, which was reported to quantify tree canopy cover^13^. After converting MODIS VCF data to tree crown cover by dividing the percent tree cover values by a factor of 0.8 as suggested by Hansen *et al*.^13^, the global average *MD* decreased to 5.34% for vegetated areas, and the proportion of pixels exhibiting an *MD* less than 15% increased to 74.37% (Fig. 8b). From a spatial perspective, the disparities between GLOBMAP and the comparative products were generally small in densely forested regions and non-vegetated areas. For example, the *MD* was predominantly within ± 10% in tropical rainforests, and the tree cover values across all five products were approximately to zero in barren lands. Greater differences were found in regions with moderate tree cover, such as in temperate forests and southern boreal forests. The most pronounced differences were noted in sparsely tree covered areas, such as the African savannas and the northern boreal forests, where the *MD* values could exceed 20%. This suggested that considerable uncertainties persist in the estimation of low tree cover using medium-resolution remote sensing data. The estimation results may be influenced by the characteristics of the input MODIS data. In regions with high cloud cover, such as the equatorial west of the Congo Basin, cloud contamination of MODIS data can result in an underestimation of tree cover when compared with the GFC, CGLS-LC00, and AVHRR VCF datasets.

### Application to global and site-scale tree cover changes analysis

**Fig. 9:**
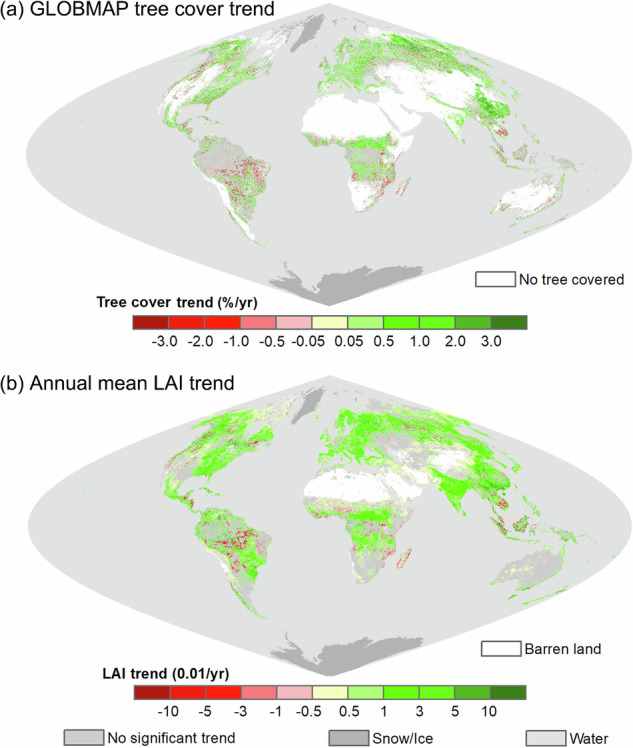
Application of GLOBMAP FTC dataset for global tree cover change analysis during 2000–2021. (**a**) Tree cover trend; (**b**) annual mean LAI trend as an indicator of vegetation status changes.

The GLOBMAP FTC dataset was applied to analyse global tree cover changes. Linear trends of fractional tree cover from 2000 to 2021 were regressed and depicted in Fig. 9a. Additionally, the linear trend of the annual average leaf area index (LAI) from 2000 to 2021 was also calculated using the GLOBMAP LAI dataset (Version 3)^39^, serving as an indicator of vegetation status changes (Fig. 9b). The GLOBMAP FTC dataset indicated an overall increase in the total area of global tree covered regions during the 22-year period. Widespread tree cover gains were observed in regions such as East Asia, the northern boreal forest zones, African savannas, and the eastern coast of Australia. The most pronounced tree cover growth was located in southern and northern China, with a rate exceeding 2% per year. In contrast, tree cover loss was mainly concentrated in the tropical zone, including the eastern and central parts of South America, the Amazon basin, central and eastern Africa, and Southeast Asia. Some areas of boreal forests in high-latitude of the Northern Hemisphere also experienced decline in tree cover. The tree cover change patterns illustrated by the GLOBMAP FTC dataset were consistent with LAI trends. Regions undergoing increases in tree cover also generally exhibited rises in LAI, suggesting that forest recovery and expansion contributed to vegetation greening in these areas. Conversely, in agricultural areas, such as India, North China Plain, and the central United States, LAI greening should mainly be attributed to crop cultivation^40,41^.

**Fig. 10:**
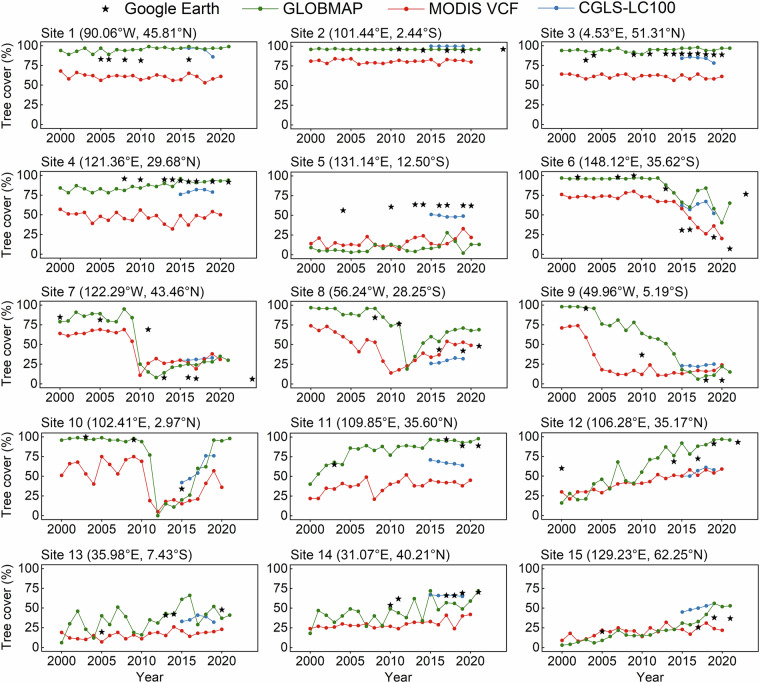
Application of the GLOBMAP dataset for tree cover change analysis at site level. These include relative stable tree cover at sites 1–5, tree cover loss at sites 6–10, and tree cover gain at sites 11–15. The tree cover percentage was visually interpreted using sub-meter-resolution Google Earth images as reference data. Similar hundreds-meter-resolution annual tree cover products were also presented, including MODIS VCF and CGLS-LC100.

To demonstrate the performance of the GLOBMAP FTC dataset in depicting forest change at the site scale, tree cover series from 2000 to 2021 were extracted for 15 sites. These sites were randomly selected worldwide for three types of tree cover changes, including remaining stable, loss, and gain, with five sites assigned to each category. For each site, all available Google Earth sub-meter-resolution images since 2000 were collected and visually interpreted to generate reference data for evaluating the dataset’s performance. To eliminate the impact of positional mismatches of our dataset and the Google Earth images, the comparison was conducted at an area of 750 m for each site, equivalent to 3 × 3 pixels in the GLOBMAP FTC dataset. For each Google Earth imagery, a 25 m-resolution grid mesh was constructed within a 750 m × 750 m area centered on the site. Grid cells dominated by trees were determined as forests, and the proportion of these forested grid cells served as the reference data for tree cover. Figure 10 illustrated the series of mean tree cover for 3 × 3 pixels around each site and the Google Earth reference data. The figure also presented the sequences of two products with similar spatial and temporal resolutions, MODIS VCF and CGLS-LC100. The results showed that the GLOBMAP FTC dataset captured the patterns of tree cover change at most sites. For sites with stable tree cover (sites 1–5), our estimation results were generally consistent, especially at sites 1–3 that were characterized by dense forests. At site 4, the estimates showed a slight upward trend in tree cover from 2000 to 2015 and then stabilized, likely reflecting the slow growth in tree crowns observed in satellite observations. At site 5 with medium tree cover, the estimates showed no significant changes but were underestimated. For the sites experiencing tree cover loss (sites 6–10), this dataset successfully captured both sudden or gradual declines in tree cover, and its annual data can represent the year changes occurred and track the recovery process. For sites with tree cover gain (sites 11–15), despite uncertainties and fluctuations in the retrievals at medium and low coverage, the dataset was able to capture the gradual upward trend in tree cover.

## Usage Notes

This study presents a global annual fractional tree cover dataset (GLOBMAP FTC) for the period from 2000 to 2021 with a spatial resolution of 250 m. This dataset can characterize variations in forest density by quantifying the proportion of dense forest within each pixel. It can support the monitoring of forest cover dynamics, not only the abrupt forest loss but also the gradual changes associated with forest recovery and expansion, which could facilitate the monitoring ecosystem restoration. We realigned global annual satellite observations to a common phenology and condensed the data into twelve features to extract tree cover. This greatly reduced the feature dimension of the model compared to conventional global tree cover mapping, which helped prevent model overfitting and improve the stability of retrievals^42^. Besides, a massive training dataset comprising over 465 million sample points across the globe was collected to train the neural network, which would enhance the model performance. This dataset can be applied to assessment of the progress of ecological initiatives, such as the United Nations Decade of Ecosystem Restoration, and various afforestation and forest conservation projects. It can also be used for the monitoring of forest distribution and status, which are crucial for ecological research and forest management. Furthermore, this fractional tree cover dataset would contribute to improve forest ecosystem modelling, given the distinct photosynthetic capabilities, carbon cycle mechanisms, and ecosystem functions provide by trees in contrast to herbaceous vegetation. The uncertainties of the dataset were primarily related to the potential overestimation of tree cover in the training data and the difficulty of coarse MODIS observations in depicting sparse and fragmented trees. It may overestimate medium tree cover and may not be able to detect extremely sparse tree cover due to its limited spatial resolution. This challenge is particularly pronounced in regions with scattered and fragmented trees such as urban areas, agricultural landscapes, and arid zones. Caution is therefore needed when applying this dataset in these areas. In the future, high-resolution satellite data from Sentinel-2 and Landsat could help to enhance the tree cover extraction in these areas.

## Data Availability

The code of data processing and tree cover mapping algorithm are available on GitHub (https://github.com/Tamako2024/FNN_forest).
